# Healthcare staff’s perspectives on long-acting injectable buprenorphine treatment: a qualitative interview study

**DOI:** 10.1186/s13722-024-00458-6

**Published:** 2024-04-05

**Authors:** Johan Nordgren, Bodil Monwell, Björn Johnson, Nina Veetnisha Gunnarsson, Andrea Johansson Capusan

**Affiliations:** 1 Department of Social Work, Malmö University, Nordenskiöldsgatan 1, 211 19 Malmö, Sweden; 2 Department of Social Work, Jönköping University, Jönköping, Sweden; 3 School of Social Work, Lund University, Lund, Sweden; 4 Center for Social and Affective Neuroscience, Department of Biomedical and Clinical Sciences, Linköping University, Linköping, Sweden; 5 Psychiatric clinic, County Hospital Ryhov, Jönköping, Sweden

## Abstract

**Background:**

Long-acting injectable buprenorphine (LAIB) formulations are a novel treatment approach in opioid agonist treatment (OAT), which provide patients with a steady dose administered weekly or monthly and thus reduce the need for frequent clinic visits. Several studies have analyzed patient experiences of LAIB but the perspective of OAT staff is unknown. This study aimed to explore how healthcare staff working in OAT clinics in Sweden perceive and manage treatment with LAIB.

**Methods:**

Individual qualitative interviews were conducted with OAT physicians (n = 10) in tandem with nine focus group sessions with OAT nurses and other staff categories (n = 41). The data was analyzed with thematic text analysis.

**Results:**

Five central themes were identified in the data: (1) advantages and disadvantages of LAIB, (2) patient categories that may or may not need LAIB, (3) patients’ degrees of medication choice, (4) keeping tabs, control and treatment alliance, and (5) LAIB’s impact on risk and enabling environments in OAT. Overall staff found more advantages than disadvantages with LAIB and considered that patients with ongoing substance use and low adherence were most likely to benefit from LAIB. However, less frequent visits were viewed as problematic in terms of developing a treatment alliance and being able to keep tabs on patients’ clinical status. Clinics differed regarding patients' degrees of choice in medication, which varied from limited to extensive. LAIB affected both risk and enabling environments in OAT.

**Conclusions:**

LAIB may strengthen the enabling environment in OAT for some patients by reducing clinic visits, exposure to risk environments, and the pressure to divert medication. A continued discussion about the prerequisites and rationale for LAIB implementation is needed in policy and practice.

**Supplementary Information:**

The online version contains supplementary material available at 10.1186/s13722-024-00458-6.

## Introduction

Long-acting, weekly or monthly, injectable depot buprenorphine (LAIB) formulations in the treatment of opioid use disorder reduce the need for daily supervised dosing, enabling clinicians to provide medication with similar efficacy as with sublingual dosing [1, 2]. LAIB results in lower healthcare service attendance and entails practically no risk for diversion [2]. LAIB thus has the potential to reduce the treatment burden for both patients and clinicians, for example by reducing the time-consuming activity of monitoring sublingual buprenorphine administration [3]. LAIB has been described as a more convenient and flexible treatment option in which longer treatment intervals allow patients to visit opioid agonist treatment (OAT) clinics less frequently compared to the “traditional” OAT structure [4]. This flexibility might mean more time available for patients to work, study and distance themselves from drug use subcultures [5].

When asked about their experiences of LAIB, patients report a sense of freedom, stability and normalcy, based on not having to go through a short-temporal daily dosing cycle [3, 5, 6]. However, the longer interval may disrupt engagement with psychosocial and practical support offered at clinics [5], which can be felt as a loss of social interaction and daily routine [3]. The sense of freedom to visit a clinic weekly or monthly instead of daily, tethered with a sense of loss of social connection and informal care offered by the clinic, is a dilemma in LAIB treatment [3, 6]. OAT clinics offer a range of medical treatment interventions, psychosocial support, and sociality both between patients and staff and between patients, enabling health and well-being. At the same time, OAT clinics may be understood as risk environments [7], where patients may face stigmatization and control, or encounter other patients who sell illicit drugs or who act in threatening or violent ways [8]. Such aspects of a risk environment can jeopardize patients’ desire and opportunity to move towards a more stable treatment phase and life situation.

Studies of LAIB focusing on patients’ experience have shown high patient satisfaction in reducing drug craving, withdrawal symptoms and self-reported reduction in ongoing illicit drug use [9]. LAIB may break a daily ritual of taking sublingual tablets and significantly decrease the number of clinic visits [10]. Patients report a preference for monthly LAIB compared to weekly [11], but some patients may reject LAIB because it can limit their control over dosing [5, 6]. Patients report that LAIB enables them to “pass” as people who are not enrolled in OAT, which may reduce the stigma associated with OAT and with being a person with former illicit drug use [12]. LAIB also precludes the risk of diversion of OAT medications, a risk that is a continuous concern in OAT with methadone and sublingual buprenorphine [13]. However, some patients might be reluctant to accept LAIB because it removes an opportunity for income generation when selling medication [5]. In general, LAIB may benefit patients who risk missing daily appointments, those at risk of diverting medication, [9, 10], patients living in rural areas [14], and patients incarcerated in prison [15].

In Sweden, OAT has long been regarded as controversial, with high thresholds to treatment and various limitations to treatment modalities [16]. Current national guidelines emphasize access to treatment and to harm reduction measures [17] while still mandating three months of daily supervised dosing for sublingual/oral OAT medications, which in practice can be longer for patients with low adherence and continued illicit substance use [16]. To a certain extent, there are still large regional variations both in the professional doxa of OAT clinics, and in access to OAT [18]. Implementation of LAIB also varies between clinics, since some clinics have been quick to implement LAIB while others have been slower to do so.

Due to the relatively recent introduction of LAIB, research regarding the viewpoint of healthcare staff and how this novel treatment option has been introduced in clinical practice is lacking. Although several studies have investigated patients’ perspectives on LAIB, we have not found any previously published research on professional and staff perspectives in healthcare settings. Staff are responsible for implementing LAIB and adapting OAT to these new formulations. Health care staff experience and perception of LAIB and how they consider and respond to patients’ perceptions and experiences of LAIB, will potentially influence the therapeutic alliance and staff's capacity to create a positive pharmaceutical atmosphere [19]. This study aims to explore how healthcare staff working in OAT clinics in Sweden perceive and manage treatment with LAIB.

## Methods

We employed a qualitative research strategy to study how the staff perceived and managed LAIB in their clinical practice. Semi-structured interviews were conducted individually with physicians at the OAT clinics. We also conducted focus group interviews with treatment staff, including nurses, counselors and other clinical caretakers.

### Recruitment and participants

We started with a pilot, where we invited staff from two clinics to participate (not included in the final data analysis). Because physicians tended to set the tone in the discussion, and some of the staff were reluctant to express their opinions freely, we decided to conduct semi-structured interviews with the OAT physicians and focus groups with the remaining staff from each clinic separately. All staff members who worked with treatment were invited to participate in the focus group interviews.

### Focus group interviews with OAT nurses and clinical caretakers

Focus group sessions with nurses, counselors and clinical caretakers were conducted via Zoom [20] by the second and fourth authors. In total, 41 persons attended nine different focus groups. The average length of the interview sessions was 52 min, with a range of 47–60 min. The semi-structured focus group discussion guide contained the following main topics: (1) positive and negative aspects of LAIB, (2) impact on social support, (3) factors affecting which medication was prescribed, (4), information received about LAIB, (5), the extent to which staff decide about medication choice, (6) views on support versus control in OAT, and (7) views on medication diversion.

### Semi-structured interviews with OAT physicians

Ten physicians were interviewed individually via Zoom. The average length of the interviews was 50 min (range 34–60 min). The semi-structured interview guide contained the following topics: (1) demographic information and professional background, (2) views about OAT, cooperation with other services, clinical routines, clinic “culture” and control versus support, (3) introduction and implementation of LAIB, (4) administration of LAIB (positive and negative experiences, comparison to other medications, dosage period aspects, social support), (5) patients’ and staff’s views of LAIB, and 6) views on future developments in OAT.

### Data analysis

The interview transcripts were read and inductively coded in NVivo by the first author. The coding process followed principles of thematic text analysis [21, 22]. The sorting of the transcribed material involved an initial open coding in which each phenomenon contained in the data was assigned a code that clarified its meaning [22]. In this way, a text can be broken down, processed, conceptualized and split into categories, with the aim of discovering categories that capture the fullness of the experiences and actions that are studied [21]. This initial coding process resulted in 52 categories relating to understandings and practices regarding LAIB among OAT clinic staff, such as: “Reduced insight about the patients by staff” and “A sense of security for staff”.

The categories were subsequently discussed by all authors to reach consensus about which to include in the thematic analysis. Authors two and three conducted an additional full coding of the data as a validation to the initial coding. After consensus on relevant categories was achieved, the first author conducted an additional analysis of the categories with the aim of identifying themes and subthemes. The criterium for theme inclusion was that it should capture a prominent aspect of the data in a patterned way, in alignment with the research questions [21]. The themes were then discussed by all authors and a final decision on inclusion of themes was taken. In the final analytical step, quotations that exemplified and illuminated each theme and subtheme were selected. The quotations were then translated from Swedish to English by the first author (Additional file 1).

## Findings

The participants, namely physicians (n = 10), and treatment staff (nurses, counselors, and other clinical caretakers) (n = 41), were recruited from 10 OAT clinics in Sweden. Clinics were situated in various parts of Sweden, including both large cities, and rural and small-town areas. Distribution between the sexes was eight men and two women among the physicians, while nurses and clinical caretakers were mainly female (34 women and 7 men). The participants had worked with OAT for an average of 7.0 years; physicians for 6.9 years (range 1–17 years), nurses and clinical caretakers for 7.3 years (range 1–22 years).

In the following we present the five themes identified in the data. We first present advantages and disadvantages of LAIB as assessed by the staff. We then turn to how staff discussed different patient categories that may or may not need LAIB. Next, we report on degrees of choice in medication and how staff viewed keeping tabs on patients, aspects of control and treatment alliance. Finally, we present how staff viewed LAIB's impact on risk and enabling environments in OAT.

### Advantages of long-acting injectable buprenorphine

The staff reported a wide range of advantages with LAIB and viewed LAIB as a valuable treatment option in OAT. The most mentioned advantage was that patients do not have to come to the clinic as often as they used to, which at least in theory open new possibilities of working or studying. Increased stability in terms of having an even buprenorphine dosage compared to the sublingual dispensation was another advantage that several interviewees mentioned. Another advantage expressed by the interviewees was that LAIB enables staff to feel more secure about patients using their medication as intended. One interviewee described this as the patients having a “buprenorphine-protection in them” (Participant 11, nurse) and a physician described it as having an “overdose protection” (Participant 10, physician).

This aspect of “protection” was often coupled with the pharmacology of buprenorphine with high-affinity binding to the mu-opioid receptor, which gives relative protection against overdose with full agonists with lower receptor affinity (such as heroin or methadone). It was also coupled with the issue of diversion and the ability to trust patients with their medications, as exemplified by the following quote from a physician:

/…/ the nice thing about the injection is that you never have to think about if the patient has an ulterior motive. /…/ Otherwise, you always have to keep in mind if the patient wants to sell surplus or if he’s honest about having too small a dose. /…/so you can trust the patient, which you couldn’t before, to be honest (Participant 3, physician).

Reduced diversion was another aspect mentioned, as illustrated by the following quote: “It feels good as a prescriber to know that the medicine ends up where it should. /…/ We simply remove the risk that they [patients] misuse their medicines” (Participant 6, physician). The staff also mentioned advantages related to patients moving away from a mindset of “wheeling and dealing” medicines. Also, by reducing the need for control, LAIB free up time for other work-related tasks among staff.

### Disadvantages of long-acting injectable buprenorphine

The main disadvantages described by the interviewees were: a reduced overview of the patients, reduction in social support to patients, physical side effects, and patient experiences that LAIB do not fully treat withdrawal symptoms.

Some staff reported that LAIB had a shorter effect duration than the expected week or month. Patients reported withdrawal symptoms and had to take the next injection earlier than expected. This was most common in the induction phase, but a few interviewees also reported that the problem persisted, as in the following excerpt:

With [LAIB] specifically there was a difficult period in the beginning for the patients because they end up in withdrawal in the initiation phase or risk going into withdrawal. So, a little bit of craftmanship is needed to minimize risks. Very clear information to the patients is also needed so that they know what to expect. We put much effort into doing that. But otherwise I think that, sometimes it may be that even later, after induction, they don’t really last four weeks and then you have to give an injection earlier (Participant 8, physician).

Physical side effects reported by patients to staff included lumps, rashes, pain or aching at the injection site, abscesses, feeling intoxicated, feeling depressed and tired, and swollen arms and legs, in line with previous research on patient experiences [6, 23]. In some cases, the experienced side effects were so severe that patients had to switch to another depot formulation, or back to tablets.

Staff reported that patients have rituals surrounding their OAT medication and that some found it hard not being able to adjust their dosage themselves, as indicated in the following exchange from one of the focus groups:

Several, but not all, have said that there’s a kind of ritual with the tablets. They’re used to doing it a certain way every morning. And some split the dose and they all do it differently. […] With the injection [the effects] becomes featureless. And that is what you want with an even concentration. But they are used to things happening. […] (Participant 27, nurse).

Loss of control I would say. That they don’t have the possibility to control their intake themselves. I think many are bothered by that (Participant 28, nurse).

For some patients this aspect was important enough to make them quit LAIB, as indicated in the following quotation:

In our experience those who get a lot of anxiety cannot cope with making this transition: ‘Oh I’m not allowed to take this, I must have my tablet, it’s so psychologically important to me’. They cannot handle this deprogramming. It’s usually those patients that quit LAIB (Participant 8, physician).

Although these disadvantages were discussed in detail by the interviewees, it is worth noting that the overall experience of staff was that the advantages of LAIB outweighed the disadvantages.

### Patient categories that may or may not need LAIB

The different clinics made somewhat different assessments about which types of patients LAIB is most suitable for. Although the overall view was that initiation of LAIB must be based on individual assessment of patients, we found a dividing line between the categories of so-called “well-functioning” and “harm reduction” patients. It should be noted however that the issue of prioritization was complex, since some of the clinics faced local, non-medical, economic or administrative restrictions regarding the number of patients to whom they could offer LAIB.

#### ”Harm reduction” patients with complex needs

Patients with complex needs, described by the staff as “harm reduction” patients was a patient category with more severe problems, discussed by staff as engaging in illicit drug use, having difficulties in coming to appointments, and experiencing psychiatric comorbidities.

The majority of the clinics in the sample had decided or had learned over time that LAIB was best suited for this category of patients. The main motive was to help these patients to adequate medication, which also sustained a sense of patient safety among staff. Initially, as one nurse explained, “well-functioning” patients who had progressed well in treatment, were thought to have the most need for LAIB:

Initially, we thought that it was a formulation that we would give to patients who were in a state of rehabilitation, and that they needed it to be able to work and so on. But we discovered that it might save patients who were in a really bad situation (Participant 14, nurse).

A physician said that they mainly used LAIB for patients with low treatment adherence: ”We mostly use it if there are significant problems with adherence and if they don’t come [to the clinic]” (Participant 10, physician). There were a small number of cases in the data when LAIB had been used for patients that were seen as threatening, as in the following example:

Some who had made threats, I think, where they didn’t want them at the clinic that often. It was because they were acting in a threatening manner in meetings and being really disruptive at the clinic. That’s why they gave them weekly or monthly injections to make them come less frequently (Participant 5, physician).

Although this was an infrequent aspect in the data, it is a novel finding that we have not seen reported before.

The staff discussed ambivalence related to the challenges of monitoring the situation of patients with complex needs, when meeting them less frequently:

We have used [LAIB] to a very high degree to be able to help patients who have low adherence. /…/ I think [LAIB] has been a revolution in treatment for these patients, but I also think that there is uncertainty about the situation of the patient when they are away [from the clinic] for a month (Participant 11, nurse).

Overall, the discussion revolved around the dilemma of keeping tabs on “harm reduction” patients with complex needs in terms of medical risks in relation to less frequent clinic visits. One of the physicians avoided introducing LAIB to this patient category:

Our main concern is medical safety. Because if the patient gets sublingual treatment and comes to us daily then we see day-to-day what shape they are in and then we can quickly alert the social services or call an ambulance or send them to detox. But if they are away for a whole week or even worse a whole month and they have no reason to come to us, then we lose the possibility to act swiftly (Participant 7, physician).

#### “Well-functioning” and stable patients

Patients with high adherence were defined as “well-functioning” or “stable” and were described as working or studying. These patients commonly picked up their medicines at pharmacies and generally visited the OAT clinics seldom. A minority of the clinics prioritized LAIB for patients with high adherence to treatment. One physician talked about patients for whom his clinic would prefer to use LAIB: ”Let’s say that they have a job or the possibility to get a job or to study. Then we would push a bit more for [LAIB] and for the possibility to get that” (Participant 5, physician). This physician elaborated further, saying that “well-functioning” patients were prioritized either by staff or because the patients themselves had requested LAIB:

It has been for relatively well-functioning patients that still have jobs where we have recommended it on some occasions, or if they themselves have asked for it because they have heard about the treatment. Where we can avoid them having to come to the clinic every day for the first three months and instead come once or twice per week. In the beginning [they come] twice per week because we have to give the injections more frequently but also because we want to follow up on how they feel (Participant 5, physician).

Staff often motivated prioritization of LAIB to “well-functioning” patients based on increased possibilities to facilitate work or studying or to encourage patients to seek employment or education.

Another reason for offering LAIB to “well-functioning” patients was to reduce the amount of take-home medicines for patients who seldom visited the clinic or who picked up their medicines at pharmacies. However, infrequent clinic visits were also mentioned as a reason not to offer LAIB to this patient category:

For a pharmacy patient who only comes to the clinic a couple of times each year [LAIB] results in an increased frequency to come here. It might even mean that they feel more bound up [to the clinic] in a negative way (Participant 8, physician).

Overall, we found that some patients with high adherence could be considered for LAIB, based on their need for fewer visits due to other obligations such as work or study. However, there was a breaking point at which depot injections would actually increase the number of visits.

### Patients’ degrees of medication choice

The extent to which patients were allowed to freely choose whether to initiate LAIB or not differed between the clinics. The overall view was that patients were allowed to choose in cooperation with staff, but some clinics had forced some patients to initiate LAIB. Also, some clinics operated under economic or administrative restrictions limiting access to LAIB.

#### Extensive choice

Most clinics had a clear aim to allow as much patient choice as possible and would discuss different options with the patients. In the following excerpt, a physician discusses the extent to which patients were allowed to make a choice regarding medication:

Interviewer:

To what extent can the patient influence the choice of medicine?

They can influence it quite a lot. I don’t think it will be a good treatment if you force the patient to take something they don’t want. Exceptions are… They will get Suboxone if they use illicit drugs and when I have a really big suspicion about diversion of doses. Then I use either depot or Suboxone and I’m restrictive with mono buprenorphine. But otherwise, I try to listen to what the patients want (Participant 7, physician).

In Sweden, since 2015 mono buprenorphine has had a low priority as first-choice medication for OAT in the national guidelines. One clinic was in the process of phasing out mono buprenorphine, and in that case, non-pharmacy patients treated with buprenorphine would have to choose between either a buprenorphine-naloxone combination or LAIB.

#### Limited choice

At one of the clinics, staff were open to the fact that some patients had been forced to initiate LAIB. This was seen as problematic by the interviewees, as exemplified in the excerpt below:

It [LAIB] having been forced on some patients can make them feel rather powerless and as not having the possibility… before everyone had the possibility to take part and guide their own treatment but then there was a rather big shift about that. ‘Now we have [LAIB] and you can no longer decide for yourself but we will decide for you’ (Participant 21, nurse).

This clinic stopped using mono buprenorphine and forced those patients to start LAIB. This was described by one nurse as problematic both in terms of an exercise of power, but also since it increased the staff workload:

All patients had to try [LAIB], whether they wanted to or not. So, it was kind of enforced. Staff became split about that and, also toward the patients. It became tiresome for the staff to have to take on these battles as well. And for the patients, who usually also have their own willpower, it became extreme and painful to all the time have to work actively, pushing the patients to switch formulation. To some extent, I think it meant that it resulted in an increase in staff sick leave (Participant 21, nurse).

The overall tendency in the data was that “harm reduction” patients with complex needs were allowed a lesser degree of choice, as exemplified in the following quotation:

We might end up in a situation where the patient takes a lot of heroin mixed with a whole lot of other substances and where they don’t have the ability to come to the clinic. And where the situation might be that we say: ‘Well, either you take [LAIB] or nothing”. And then that is not to have a choice (Participant 11, nurse).

We found that the degree of choice for patients on whether to initiate LAIB or not varied between the clinics in the study, from an explicit patient choice perspective and shared decision making to a more enforced demand from staff to patients.

### Keeping tabs, control, and treatment alliance

A central theme in the interviews concerned the importance staff attached to keeping tabs on the patients and having knowledge about their current status. Several interviewees saw weekly and monthly meetings as a significant challenge, compared to daily clinic visits during treatment initiation with sublingual buprenorphine or methadone. In the following quote a nurse describes a sense of loss of both a treatment alliance and of being updated about the patients’ lives and progress in treatment:

For tablets they must come daily for the first three months. When you meet someone at least five days per week, you get to know them well and know what it looks like at home and what kind of relationships they have with their family, and… friends and if they have a job or not. You develop quite a close bond. But you lose a lot of that when they have the injection. And then you cannot keep tabs the way we would like to (Participant 30, nurse).

Another nurse said: ”I think that you miss quite a lot when you don’t meet the patient more than perhaps once a week. I think that what is a freedom for them turns into a disadvantage for us sometimes” (Participant 29, nurse). Less frequent visits were described as challenging from a medical safety standpoint, as in the following example:

You don’t know exactly what they do between the visit days. I’ve had a patient who got LAIB quite quickly and where it turned out that… several months passed before we found out that this patient was testing positive for cocaine sometimes. So, when you don’t meet them that often, you don’t get to know what’s happening between the meetings (Participant 36, nurse).

Control can also be regarded as an exercise of power. The following excerpt is an isolated one in the data, but exemplifies how some staff might view LAIB as reducing their ability to “punish” patients who use illicit drugs:

There were problems in the beginning, a lot of discussions about ‘this patient will have to go back to tablets’. There was uncertainty among staff, particularly nurses. They lost the possibility to teach a lesson. You cannot reduce the dose when they use illicit drugs. That possibility disappeared and the nurses did not appreciate that (Participant 2, physician).

This was an initial reaction to LAIB, but the quote reveals how power dynamics between staff and patients in OAT may influence the introduction of a new treatment option.

Although a minor theme, another aspect of the treatment alliance concerned the way LAIB might increase the workload of staff. Less frequent meetings with more patients can increase the number of requests for support from patients. This aspect was commented upon by one physician:

It’s a bit surprising, because I thought that they would be thankful to get rid of the daily contacts and instead meet more patients who come seldom. But the opposite was true, they feel that the workload is heavier, because the patients who they meet daily, it’s not a big thing for them, they keep tabs on them, they know exactly in what shape they are, and they know what’s happening. But for those who come more seldom, like once per month, so much has happened since the last time and then the patients want to sit a really long time and talk and bring up all kinds of things. They want dental and medical certificates and renewed prescriptions. So it becomes a bit overwhelming to have these patients (Participant 7, physician).

Particularly among staff such as nurses, who often meet patients daily, the implementation of LAIB was found to negatively affect their abilities to follow up the patients’ current health and social situation. Although patients on LAIB were viewed as having a form of overdose protection, the weekly or monthly meetings for “harm reduction” patients were mainly seen as a risk that impeded the ability to act in cases of impending problematic situations, such as increased illicit drug use or worsening mental health.

### Impacts on risk and enabling environments in OAT

The way that LAIB significantly limits the diversion of other OAT medications was frequently brought up as an important advantage of the treatment option. This aspect related mostly to the possibility of ensuring that patients with low adherence used their medications as intended, without the possibility of giving, trading or selling take-home doses.

Staff also discussed the way that LAIB, by reducing the frequency of visits, can reduce the risks of patients meeting other patients or dealers who want to sell illicit drugs to them. One nurse brought up this issue in relation to the risk of meeting patients with continual drug use:

I think it’s an advantage to not come here and meet other patients who continually use illicit drugs. That can be a risk factor for them to relapse. But also, to not have to meet with the people who, unfortunately, come around here, by the clinics, and who want to sell illicit substances. If you come once per week or once per month you minimize the risk of being exposed to that (Participant 35, nurse).

A nurse brought up that patients found it relieving to have LAIB so that they could avoid being asked to sell their medications:

I have a patient who says that she has the injection even though she’s on tablets to get rid of the nagging in the waiting room that she should sell her tablets. She pats herself like this [pats the thigh] to make everyone think she has gotten an injection instead (Participant 27, nurse).

These excerpts indicate a strong sense of how staff perceived that LAIB significantly reduced risks experienced by patients when coming for daily visits to OAT clinics.

## Discussion

Our findings from focus groups and interviews with healthcare staff highlight both the opportunities and dilemmas that LAIB entail in the clinical practice of OAT. Overall, the staff perceived more advantages than disadvantages with LAIB. Advantages and disadvantages described were similar to those resulting from interviews with patients [5, 6, 11].

We found a significant dividing line in the staff’s perceptions of whether LAIB was most suitable for low versus high adherence patients. Staff described a learning process, in which they initially assumed that LAIB would be more beneficial for high adherence patients, but later found that LAIB can offer significant benefits for patients with complex needs described as “harm reduction” or low adherence patients. Both of these patient categories were positive towards LAIB and reported similar advantages and to some extent similar disadvantages [6, 24]. The notion of “harm reduction” patients as a homogenous patient category is problematic since the category is broad, and different practitioners of OAT may define and view harm reduction differently [25]. The treatment process is also dynamic, and a patient might need harm reduction interventions at some points in the process and not others. Nonetheless, the category seems to act as a proxy for problem severity and may have been useful for OAT clinics in differentiating between patients in terms of the usefulness of LAIB.

Less frequent visits made keeping tabs on the patients more difficult. According to our interviewees, this may negatively impact the staff’s ability to identify needs and respond promptly to reduce medical risks or offer social support. It can also be perceived as detrimental to the treatment alliance, especially if LAIB was offered as a first-line treatment option, when staff had not yet had the possibility to develop an alliance with the patients. This is noteworthy since the treatment alliance is not necessarily a function of daily visits. Daily visits are in fact very rare in psychiatric or addiction medicine settings. Also, historically in the Swedish setting, OAT has been described as fraught with tensions, defined by tight control, and verging on abuse of power, where positive drug screens could result in dose reductions as “punishment”, or, in the worst case, involuntary discharge and denial of further treatment [26]. That sporadic cocaine use in an otherwise well-functioning patient is seen as a problem may be related to the same Swedish OAT tradition, where abstinence and rehabilitation are often the main goals of treatment [18]. OAT clinics in Sweden differ in pharmaceutical atmosphere [6] and may to some extent have different interpretations and implementation of local and national guidelines.

It was also notable that some staff categories, such as nurses and counselors, found an increased workload because of having to handle more practical issues during weekly or monthly patient meetings instead of splitting them up on an everyday basis. Some of the increased workload could be attributed to increased number of patients in treatment during the same period. However, the issues concerning how to deliver social support with less frequency has also been raised in studies of patient experiences [3, 9] and constitutes an important point for further consideration in managing in clinical practice. As noted by Allen et al. [9], we also found that OAT staff often made sure that patients could contact the clinic outside of their weekly or monthly appointments if necessary. Nonetheless, we agree with Lancaster and colleagues about the need to make the social support component of OAT more present [3].

Staff also recurrently spoke about how LAIB reduces diversion and viewed diversion as a point of concern in OAT and as a destructive phenomenon in terms of medical risks and of damaging the legitimacy of OAT [27]. In one sense, the way that staff highlighted how less frequent visits could decrease risks surrounding relapse, drug dealing offers, violence and threats identified OAT clinics as a risk environment. Previous studies have described OAT clinics as both enabling and as risky environments. [8, 28, 29]. Our findings indicate the ways in which LAIB can be understood as affecting both the enabling and risky practices. For instance, from a medical perspective, LAIB offers “overdose protection” for “harm reduction” patients with complex needs, in an enabling way. From a nursing or social work perspective, less frequent client meetings may create a risk environment for this group. Another example is staff’s concern over the difficulties in maximizing social support and developing treatment alliances when patient contact is reduced, while being aware of the way OAT clinics become risk environments when patients are continually offered illicit drugs in connection with clinic visits [30]. LAIB may have an important role to play in enabling patients to avoid those kinds of risk situations. The findings strongly suggest that OAT staff who administer LAIB actively assess risks and possibilities with this new treatment and strategically work to reduce risks and develop the treatment toward enabling aspects for each individual patient.

LAIB and its introduction into Swedish OAT highlight some concerns about low versus high threshold treatment and the ways in which guidelines and rules are applied. In Sweden, traditional OAT has had a high degree of control through daily supervised sublingual/oral dosing, and frequent testing for illicit drugs [18, 31]. LAIB was introduced during a period when OAT access had increased due to changes in the national guidelines [17], which lowered thresholds for patients to enroll in treatment and no longer sanctioned exclusion from treatment due to noncompliance or rule-breaking. The less frequent clinic visits have in some settings resulted in new ways of working in OAT in which the previous strong notions of control are no longer possible. Staff in the present study problematized the changes mainly by reference to the reduced possibility of keeping tabs on patients. This aspect of problematizing stems from an increased worry that they will not be able to intervene when patients experience a negative development in their life situation and/or treatment and is as such related to a concern for the patients’ wellbeing. However, there is evidence that patients in OAT are highly critical of limited access to take-home doses since this impedes their possibility of finding and keeping employment, as well as being perceived as dehumanizing [30]. These aspects of limitations in access to take-home doses provide a useful point of comparison between daily supervised oral dispensation and LAIB.

### Implications for policy and practice

In this study, we found that LAIB is a useful treatment option that is appreciated by staff. Staff perceive LAIB to match the treatment needs of different patient groups and believe that patients view LAIB treatment favorably. However, our results also indicate that LAIB is not appreciated by all patients. Forcing patients to switch over from sublingual buprenorphine to LAIB may negatively influence relationships with staff and potentially the perception of effects and side effects in the implementation phase [6]. We argue that staff need to discuss the exact prerequisites and reasons for implementing LAIB in clinical practice at their unit/region and in each individual patient.

While a one-sided focus on reducing diversion may create ethical distress among staff, who must navigate between different often conflicting roles in their clinical practice, diversion remains a significant problem in OAT. Strategies that reduce diversion may help patients who experience a pressure to divert their medication and strengthen enabling aspects of OAT.

Although it was an isolated example in the data, the notion that LAIB could be seen as an obstacle to “punishing” patients who relapse by reducing medication doses highlights the unequal power dynamics operating in some OAT settings [32–34]. Patient and drug users’ unions, as well as OAT staff, may need to monitor these kinds of practices in OAT and we suggest that anti-stigma education [35] and shared decision making tools [36] should be incorporated with the clinical implementation of LAIB.

### Strengths and limitations

The present study has both strengths and limitations. Among strengths we note data collection from many different clinics, in urban and rural settings. We used data from a pilot study to optimize methods for getting information from different categories of clinical staff (doctors, nurses, assistant nurses). The interviews were conducted at OAT clinics with varying degrees of implementation of LAIB, which limits generalizability across all OAT clinics. The study was conducted in a Swedish context, with a tradition of paternalistic, controlling and rehabilitation-oriented OAT which may be another factor limiting generalizability to other contexts.

## Conclusions

Healthcare staff working with OAT in Sweden found LAIB to be more advantageous than disadvantageous, but the treatment requires a continuous discussion about which patients it is most suitable for. The long-acting nature of the medication presents a challenge for staff regarding keeping tabs on patients but may also provide new perspectives on therapeutic alliance in OAT, building on patient’s needs rather than daily or frequent supervised intake over extended periods of time. LAIB was seen as strengthening the enabling environment for most patients, while at the same time highlighting the OAT clinic as a potential risk environment especially for patients struggling with ongoing use who are not on LAIB and must visit the clinic frequently. This study provides new knowledge on OAT staff’s perspectives and strategies in working with LAIB. Further research is needed regarding the day-to-day work that is carried out in different national and regional settings.

### Supplementary Information

**Additional file 1.**COREQ checklist.

## Data Availability

The datasets generated during and/or analysed during the current study are not publicly available due to pseudo anonymity of research participants, but are available from the corresponding author on reasonable request.
