# Supplementary material for: Healthcare staff’s perspectives on long-acting injectable buprenorphine treatment: a qualitative interview study
Source: Addict Sci Clin Pract. 2024 Apr 5;19:25. doi: 10.1186/s13722-024-00458-6 (PMC10996245; doi:10.1186/s13722-024-00458-6)
Supplement: Supplementary file 1 — Additional file 1.COREQ checklist. [file 13722_2024_458_MOESM1_ESM.docx]

| **Domain 1: Research team and reflexivity** |  |  |
| --- | --- | --- |
| Personal Characteristics |  |  |
| 1. | Interviewer/facilitator | Which author/s conducted the interview or focus group?  **Focus groups: Nina Gunnarsson (NG), Bodil Mowell (BM)**  **Interview: Per Bülow (PB)** |
| 2. | Credentials | What were the researcher's credentials? *E.g. PhD, MD*  **NG: PhD; BM: PhD, PB PhD;**  **Johan Nordgren (JN), PhD; Björn Johnson (BJ) PhD, Professor; Andrea Johansson Capusan (AJC) MD, PhD, Associate Professor** |
| 3. | Occupation | What was their occupation at the time of the study?  **NG: Senior lecturer in social work**  **BM: Social worker in psychiatry and addiction medicine; lecturer**  **PB: Social worker, Senior lecturer, retired**  **JN: Senior lecturer in social work**  **BJ: Professor Social Work**  **AJC Associate professor, Psychiatry, Senior consultant psychiatry and addiction medicine** |
| 4. | Gender | Was the researcher male or female?  **Two interviewers were female (NG and BM) and one male (PB)** |
| 5. | Experience and training | What experience or training did the researcher have?  **NG, BM, JN, BJ, PB extensive experience in qualitative research, interviews and focus groups.**  **BM, PB also have clinical experience in working with patients in addiction and being part of a team.**  **AJC experience in addiction research and addiction medicine.** |
| Relationship with participants |  |  |
| 6. | Relationship established | Was a relationship established prior to study commencement?  **AJC established contacts via email with directors and doctors working at all involved clinics.**  **NG did not establish any previous contacts with the clinics, other than for scheduling the focus groups and interviews. BM previously had work-related contact with several of the physicians interviewed. No relationship with participants in the focus-groups.** |
| 7. | Participant knowledge of the interviewer | What did the participants know about the researcher? e*.g. personal goals, reasons for doing the research*  **Only that they were researchers associated with an ongoing study regarding clinical use of depot injections in opioid use disorder.** |
| 8. | Interviewer characteristics | What characteristics were reported about the interviewer/facilitator? e.g. *Bias, assumptions, reasons and interests in the research topic*  **None** |
| **Domain 2: study design** |  |  |
| Theoretical framework |  |  |
| 9. | Methodological orientation and Theory | Thematic text analysis |
| Participant selection |  |  |
| 10. | Sampling | Clinics were approached by AJC and BM via email to explore interest in participating in a focus group or interview in order to find the most convenient time for the staff to participate.  Clinics were from rural and urban areas from several areas of Sweden.  Everyone working at the respective clinic was invited to participate.  Invitations were conveyed via email to a contact person who booked the focus group for the rest of the staff.  Interviews with doctors were scheduled individually. BM contacted via email all doctors working at the clinics (seven clinics) involved in the projects.  Bodil scheduled interviews. Since BM had had previous contacts with several of the doctors involved in the project, interviews were done by PB, via Zoom. |
| 11. | Method of approach | How were participants approached? e*.g. face-to-face, telephone, mail, email*  Participant clinics were initially approached via email or phone to establish contact route and find suitable time to schedule focus group to involve as many of the staff as possible.  Some of the staff were on sick leave or did not want to participate. Participating in the focus groups was voluntary, staff did not need to motivate whether they wanted to participate or not.  Focus groups were then scheduled and Zoom link to the meeting sent out to staff. Staff had the option to sit together in a room which was connected via Zoom with the interviewers or join the meeting directly via Zoom.  Individual interviews were conducted via Zoom. |
| 12. | Sample size | Physicians: 10  Nurses/caretakers: 41 |
| 13. | Non-participation | How many people refused to participate or dropped out? Reasons?  **Focus groups:** One unit invited to participate declined due to high staff turnover / new physician.  **Interviews** – none of the contacted doctors refused to participate, however one of the participants terminated her employment during the study and the research team were unable to reach her. Another physician at another clinic was not possible to reach via mail. |
| Setting |  |  |
| 14. | Setting of data collection | Clinic |
| 15. | Presence of non-participants | Was anyone else present besides the participants and researchers?  **Focus groups: none**  **Interview: none** |
| 16. | Description of sample | What are the important characteristics of the sample? *e.g. demographic data, date*  Experience of clinical/patient work within Opioid Agonist Treatment (OAT) and long acting buprenorphine depot-injection preparation |
| Data collection |  |  |
| 17. | Interview guide | Interview guide was followed.  Pilot interviews were conducted. |
| 18. | Repeat interviews | No |
| 19. | Audio/visual recording | Yes, both. |
| 20. | Field notes | Were field notes made during and/or after the interview or focus group?  No fieldnotes made. |
| 21. | Duration | Focus-groups: Average 52 minutes, range 47-60 minutes. Range 34 to 60 minutes.  Physicians: Average 50 minutes, range 34-60 minutes. |
| 22. | Data saturation | Yes  All scheduled interviews and focus groups were performed. |
| 23. | Transcripts returned | No |
| **Domain 3: analysis and findings** |  |  |
| Data analysis |  |  |
| 24. | Number of data coders | Two |
| 25. | Description of the coding tree | Yes |
| 26. | Derivation of themes | Themes were derived from the data. |
| 27. | Software | Nvivo. |
| 28. | Participant checking | No |
| Reporting |  |  |
| 29. | Quotations presented | Participant quotations were presented to illustrate the themes / findings, each with a participant number. |
| 30. | Data and findings consistent | Yes |
| 31. | Clarity of major themes | Yes |
| 32. | Clarity of minor themes | Yes |
